# A Rare Manifestation of Infectious Mononucleosis Tonsillitis

**DOI:** 10.7759/cureus.41827

**Published:** 2023-07-13

**Authors:** Kaitlyn Rewis, Sara Yang, Agnes Hurtuk

**Affiliations:** 1 Otolaryngology - Head and Neck Surgery, Loyola University Medical Center, Maywood, USA

**Keywords:** epstein-barr virus, ebv, infectious mononucleosis, tonsilitis, hemorrhagic tonsillitis

## Abstract

The purpose of this case report is to describe a rare case of infectious mononucleosis (IM) hemorrhagic tonsillitis. Our patient presented with acute tonsillitis complicated by spontaneous tonsillar hemorrhage. This is a single case report with a literature review.

A 19-year-old male presented to the emergency department with a 10-day history of worsening sore throat, recurrent fevers, malaise, and dysphagia to solids and liquids, as well as a three-day history of epistaxis and hemoptysis. He tested positive for Epstein-Barr virus and rhinovirus. On exam, a “hot potato” voice was noted along with bilateral tonsillar edema, erythema, and hypertrophy. Both tonsils with dry blood coating and no exudates were visualized. Computed tomography (CT) imaging of the neck demonstrated subcutaneous emphysema isolated to the tonsils. Treatment consisted of intravenous antibiotics and steroids, followed by an oral antibiotic, with subsequent full resolution of symptoms.

This case illustrates a rare, severe manifestation of IM tonsillitis that radiographically can mimic other more severe soft-tissue neck infections on imaging, such as cervical necrotizing fasciitis. In patients presenting with hematemesis, hemoptysis, and/or epistaxis, along with tonsillar edema, erythema, and hypertrophy, a diagnosis of spontaneous hemorrhagic tonsillitis should be considered. The radiographic findings of soft-tissue emphysema in the deep spaces of the head and neck region may be seen in the setting of IM, mimicking other soft-tissue infections of the deep neck spaces.

## Introduction

Hemorrhagic tonsillitis is rarely seen today but was previously more recognized in the pre-antibiotic era [1,2]. It is commonly associated with acute or chronic tonsillitis but can be associated with various other conditions such as peri-tonsillar or parapharyngeal abscesses, infectious mononucleosis (IM), and necrotizing fasciitis [1,2]. Spontaneous hemorrhagic tonsillitis (SHT) is defined as continuous tonsillar hemorrhage for >1 hour or >250 ml of blood loss, regardless of the duration of bleeding [1]. Reported cases suggest an increased incidence in younger patients with controversial first-line treatment regimens. Here, we present a case of IM hemorrhagic tonsillitis, which represents a rare case of acute tonsillitis complicated by spontaneous tonsillar hemorrhage. This article was previously presented as a poster presentation at the AAO-HNSF Annual Meeting on September 12, 2022.

## Case presentation

A 19-year-old male with no significant past medical history presented with a 10-day history of worsening sore throat, recurrent fevers, malaise, and dysphagia to solids and liquids and a three-day history of epistaxis and hemoptysis. He initially presented to a college clinic, where COVID-19 and Group A Strep tests were negative. He was managed conservatively with Tylenol and ibuprofen, with no improvement in symptoms. One week later, he returned for re-evaluation and tested positive for Epstein-Barr virus (EBV) and rhinovirus. He was treated with Decadron but subsequently presented to our emergency room due to persistent symptoms.

On evaluation, the patient was tachycardiac and febrile to 103.1 F. Lab work demonstrated hyponatremia of 123, leukocytosis of 19.8, elevated C-reactive protein and erythrocyte sedimentation rate at 204 and 55, respectively, and elevated liver function tests. Blood cultures were negative. A “hot potato” voice was noted on the physical exam, along with edematous and erythematous enlarged tonsils bilaterally (grade 3+). Both tonsils with dry blood coating and no exudates were visualized.

**Figure 1:**
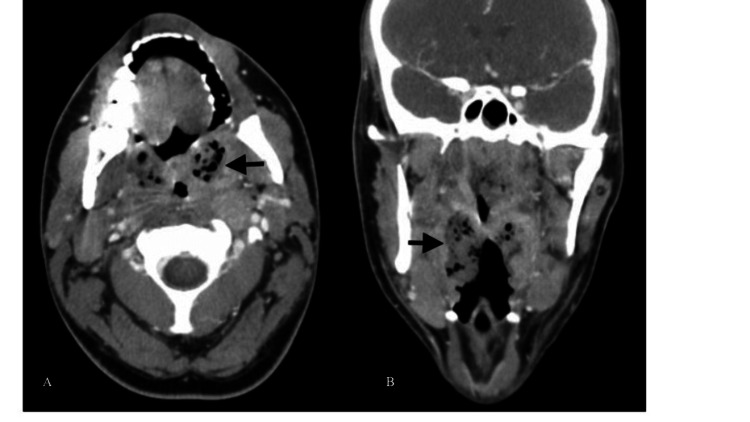
Head CT revealing soft-tissue emphysema in the tonsillar region. A) Axial view and B) coronal view CT, computed tomography.

Computed tomography (CT) imaging of the neck demonstrated hypo-attenuating rim-enhancing fluid collections within the palatine tonsils along with large amounts of air within the tonsils extending superiorly to the nasopharynx (Figure 1). Diffuse bilateral cervical lymphadenopathy was noted with no other soft-tissue emphysema present in the neck. 

Flexible nasolaryngoscopy showed inflamed nasal and pharyngeal mucosa with dried blood, but no obvious nasal or oropharyngeal source of bleeding other than the tonsils.

Diagnosis

Group A strep pharyngitis, intratonsillar abscess, necrotizing fasciitis, or infectious mononucleosis hemorrhagic tonsillitis.

## Discussion

The patient’s further blood work-up was positive for EBV, consistent with an IM infection. The bleeding in the oropharynx and the subcutaneous emphysema in the tonsil region on CT imaging appeared to be related to a raging IM infection which ultimately led to hemorrhagic tonsillitis.

IM commonly affects children and young adults and causes symptoms of fever, pharyngitis, cervical lymphadenopathy, and frequent acute tonsillitis. In rare cases, acute tonsillitis can be complicated by spontaneous tonsillar hemorrhage in the presence of IM due to increased inflammation and perfusion to enlarged tonsils [1,2]. SHT is defined as continuous tonsillar hemorrhage for >1 hour or >250 ml of blood loss, regardless of the duration of bleeding [1]. First-line treatment is somewhat controversial, ranging from local bleeding control with silver nitrate, arteriography for severe bleeding with local intervention, to emergency tonsillectomy along with antibiotic treatment [1]. Although rare, there have been reported cases of SHT in young adults and rare cases of tonsillitis complicated by massive hemorrhages resulting in sudden childhood death [2,3]. Thus, in patients who present with hematemesis, hemoptysis, or epistaxis with tonsillar pain and enlargement, SHT should be considered [2]. Our patient reported a three-day history of recurrent episodes of epistaxis and hemoptysis, a sore throat, and dry blood coating the tonsils on initial examination. Although it is difficult to quantify the total volume of bleeding, SHT was highly suspected, given the clinical history and exam findings.

The presence of bilateral hypoattenuating rim-enhancing fluid collections within the palatine tonsils can be due to inflammatory changes and tissue injury from IM versus intra-tonsillar abscesses, which do occur in IM tonsillitis. Peritonsillar/intra-tonsillar abscess formation in IM has been reported in less than 1% of EBV-positive patients and is more commonly present unilaterally with a central hypodensity and ring enhancement within the tonsillar capsule on CT scan [4,5]. A superimposed bacterial infection should be excluded, although the majority of tonsillitis cases are due to viral infections, and only 28% of patients with IM and acute tonsillitis have been reported to have positive bacterial cultures [6], with the most common bacterial infections being *Staphylococcus aureus* and *Streptococcus pyogenes* [7].

Subcutaneous emphysema in the deep spaces of the head and neck region immediately draws concern for necrotizing fasciitis. Cervical necrotizing fasciitis (CNF) associated with tonsillar abscesses is extremely rare, with only a handful of documented case reports [8]. Although uncommon, CNF should be excluded due to its rapidly aggressive destruction of soft tissues and ability to spread along fascial planes. Based on the limited case studies available, there is an apparent increase in overall mortality for patients with CNF associated with peritonsillar abscesses [8,9]. CT scan is the preferred imaging modality for diagnosis as it would demonstrate diffuse soft-tissue edema [8]. Given the lack of significant soft-tissue neck edema, no palpable crepitus on the exam, and the overall nontoxic appearance of our patient, the clinical suspicion for CNF was low.

Our patient had a laboratory-confirmed IM infection complicated by hemorrhagic tonsillitis, which was the source of subcutaneous emphysema isolated to the tonsils seen on CT imaging. The treatment course consisted of Decadron 8 mg and intravenous Clindamycin 900 mg for 24 hours with close clinical monitoring for any worsening respiratory distress or progression of infection. The patient was discharged home on hospital day 10 on a seven-day course of oral Clindamycin. This case illustrates one rare but certainly possible severe manifestation of a raging IM tonsillitis, which can mimic other more severe soft-tissue infections of the neck, such as CNF.

## Conclusions

SHT is a rare and potentially life-threatening condition where early recognition and management are essential. Although it is commonly a complication of acute and chronic tonsillitis, other infectious etiologies must be considered. Hemorrhagic tonsillitis should be considered in the differential diagnosis of a patient presenting with throat pain along with hematemesis and hemoptysis. Our case report illustrated how hemorrhagic tonsillitis could be a rare and unique manifestation of severe IM tonsillitis.
